# The capacity to work puzzle: a qualitative study of physicians’ assessments for patients with common mental disorders

**DOI:** 10.1186/s12875-018-0815-5

**Published:** 2018-07-30

**Authors:** Monica Bertilsson, Silje Maeland, Jesper Löve, Gunnar Ahlborg, Erik L. Werner, Gunnel Hensing

**Affiliations:** 1 Department of Public Health and Community Medicine, Institute of Medicine/Epidemiology and Social Medicine, The Sahlgrenska Academy, University of Gothenburg, PO Box 453, SE-405 30 Gothenburg, Sweden; 2 Institute of Medicine/Occupational and Environmental Health, The Sahlgrenska Academy, University of Gothenburg, Gothenburg, Sweden; 3 Institute of Stress Medicine, Region Västra Götaland, Gothenburg, Sweden; 4 Uni Research Health, Uni Research, Bergen, Norway; 5 Department of General Practice, Institute of Health and Society, University of Oslo, Oslo, Norway; 6 Institute of Occupational Therapy, Physiotherapy and Radiography, Department of Health and Social sciences, Western Norway University of Applied Sciences, Bergen, Norway

**Keywords:** Common mental disorder, Sickness absence, Work capacity assessment

## Abstract

**Background:**

Entitlement to sickness benefits is a legal process requiring health-related reduced work capacity confirmed by a physician via a sickness certificate. However, there is a knowledge gap concerning physicians’ clinical practice of work capacity assessments for patients with common mental disorders (CMD). Physicians claim more knowledge and skills in how to actually do the assessments. The aim of this study was to explore physicians’ tacit knowledge of performing assessments of capacity to work and the need for sickness absence in patients with depression and anxiety disorders.

**Methods:**

We performed a qualitative study with open-ended interviews and a short video vignette of a physician and a patient with depression as stimuli. Participating physicians (*n* = 24) were specialized in general practice, occupational health or psychiatry and experienced in treating patients with depression and anxiety. Interviews were audio-recorded and transcribed verbatim. Inductive content analysis was used as the analytical tool.

**Results:**

Five categories were identified. Category 1 identified work capacity assessment as doing a jigsaw puzzle without any master model. The physicians both identified and created the pieces of the puzzle, mainly by facilitating strategies to make the patient a better supplier of essential information. The finished puzzle made up a highly individualized comprehensive picture required for adequate assessment. Categories 2–4 identified the particular essential pieces of information the participants used, relating to the patient’s disorder, capacity in the work place and contextual everyday life. For the sickness absence assessment, apart from decreased work capacity, the physicians also took particulars of the work place into account; e.g. could the work place handle an employee with reduced capacity.

**Conclusions:**

Physicians’ tacit knowledge of assessing work capacity and the need for sickness absence for patients with CMD was identified as doing a jigsaw puzzle. The physicians became identifiers and creators of the pieces of the puzzle using a broad palette of essential information. Our findings contribute to the knowledge gap on clinical assessment and can be used as an educational tool. Because they are based on the professions’ tacit knowledge, acceptance of the model can be expected to be high.

**Electronic supplementary material:**

The online version of this article (10.1186/s12875-018-0815-5) contains supplementary material, which is available to authorized users.

## Background

Physicians experience assessments of patients’ work capacity in cases of sickness absence as difficult [1–4]. In a large Swedish survey among physicians, as many as 79% reported difficulties with these assessments [5]. Qualitative studies among physicians have found that the concept of work capacity is experienced as vague, often leading to difficulties in what and how to assess work capacity [6, 7]. Nielsen et al. (2011) [8] investigated decision making in the sickness certificate process and found that work capacity was almost never discussed among the physicians; this may be due to physicians’ greater interest in symptoms or lack of training in work capacity assessment. Lack of competence to assess work capacity has been identified in a narrative review as a barrier to good sick-listing practice [3]. However, few studies have explored these difficulties and none have reported how physicians actually do the work capacity assessment in more detail.

The work capacity assessment is crucial in sick-listing practice, which is a common practice, particularly in the primary health care. One Swedish study found that sickness certificates were issued in 12% of all patient encounters [9]. An English study found that one third of all issued sickness absence certificates to working patients concerned common mental disorders (CMD) [10] and Mallen et al. (2011) that sickness absence was subjected in every third case of mental health issues [11]. For CMDs particularly, physicians find the extent of the impact of CMD on work capacity more problematic to assess than for other disorders [5, 12]. Difficulties assessing whether continuing to work improves or worsens the CMD further complicates this work [4]. Compared with other cases, physicians seem to broaden their view in cases of CMD and include conditions outside work [4]. In a comparison of cases of psychological and musculoskeletal complaints, Foley et al. [13] found physicians made more inquiries about family support, the patient’s relationships and financial troubles in cases with psychological complaints. Furthermore, Wheat et al. (2015), using audio-recorded authentic encounters of sickness certificate practices found that physicians acted differently towards patients with CMD compared to other patients; taking more of a gatekeeper standpoint to these patients and being less affirmative to sickness absence [14].

Physicians’ clinical experience seems to play an important role in these assessments [3, 15]. In Sweden, a large study concluded that physicians mainly learn about sickness certification practice from experiences with colleagues and patients [16]. Furthermore, a recent review concluded that there was a lack of systematic tools and procedures to support physicians in their clinical practice [15]. Therefore, Krohne et al. [17] suggested that work capacity assessment may be “tacit assessment”, something “being in the back of the physicians’ mind” and a “gut feeling”. Clinical experience is often referred to as tacit or implicit knowledge, which is considered to be experience-based and practical, obtained through practice and repeated actions [18, 19]. In a systematic review on determinants for sick-listing patients with low back pain, it was reported that fear avoidance and distress about the complexity of unspecific pain among the physicians increased the likelihood that they issued a sick note [20]. Also the patient’s ability to evoke sympathy from the physician has been reported to have an impact on the decision [8]. In addition, general practitioners (GPs) report using negotiation strategies in their communication with patients who ask for a sick note [8]. To our knowledge, no study has explored physicians’ tacit knowledge of work capacity assessment in particular. The transition from tacit or implicit knowledge to explicit knowledge is important in order to support younger physicians to more rapidly embrace this professional skill so significant in welfare states. A first step from implicit to explicit knowledge is to verbalize the experience and know-how of practicing professionals in a systematic manner [18, 21].

The far-reaching legal and economic consequences of sickness certificates for individuals, employers and society highlight the need to explore the subject of physician assessment of capacity to work. In Sweden, an extensive evaluation found that almost half of issued sickness certificates were of low quality beyond legal standards [22]. Because CMDs have a large negative effect on work capacity [4, 23–25], a systematic and adequate assessment of work capacity is significant. CMDs are one of the most common causes for sickness absence in western societies, making it even more important to address this issue [26, 27]. The aim of this study was to explore physicians’ tacit knowledge of what and how they assess capacity to work and the need for sickness absence in patients with depression and anxiety disorders.

### Sickness absence regulations in Sweden

The first 7 days of a sick-leave spell is self-certified in the Swedish sickness insurance scheme. A sickness certificate for part-time (25%, 50% or 75%) or full-time sickness absence, issued by a physician, is required from the eighth day. Any physician, irrespective of specialty or time in service, can issue these certificates. Certification practices are handled within the clinical appointment, ranging in time between 15 and 60 min depending on health care setting. The Primary Health Care often have the shorter duration, but with possibilities to extended appointments. The clinical evaluation is in many cases facilitated by information in medical records and other sources. In this study this type of information is regarded as explicit knowledge not specifically explored further. In Sweden, patients have a freedom of choice to choose or re-choose any Primary Health Care setting. For Occupational Health Care, the patient’s work place need to have an agreement, and for Psychiatric out-door clinic care, patients most often need a referral.

Insurance physicians in Sweden only have an advisory role in the Swedish Social Insurance Agency (SSIA). In 2008, national decision support for insurance medicine was introduced aimed at standardizing diagnostically based recommendations for sick-leave length and level. There was a mixed reception for decision support among physicians. On the one hand, it was welcomed in clinical situations with clear recommendations on, e.g. length of sickness absence, and as an instrument for negotiations with the patients [28]. On the other hand, it left out important parts of physicians’ clinical management and adaptation to individual circumstances, regarded as important in the overall treatment of patients [29]. The decision support emphasizes that work capacity and sickness absence are to be assessed individually, however physicians must document the reason for durations longer than the recommendations.

In the sickness certificate the physician ought to report 1) the disorder(s) or symptoms, 2) the patient’s dysfunctions due to the stated disorder(s) and 3) the patient’s activity limitations (the consequences of the disorder(s) and related dysfunctions) which means the patients’ decreased capacity to work in relation to his/her work and work tasks. This procedure is called the DFA-chain regulation (disorder, function, activity).

## Methods

This study is part of the research program New Ways – mental health at work, aimed at identification, treatment and support for persons with CMD to remain in work.

### Design

A qualitative study design with open-ended interviews and a short video vignette as stimuli for the subject were chosen as appropriate. The study was approved by the Regional Ethical Review Board in Gothenburg, Sweden (Dnr 395–14) and the COREQ checklist was considered [30]. Participation was based on informed consent and participants were informed that they could withdraw from the study at any time. No incentives were offered.

### Setting and participants

**Fig. 1:**
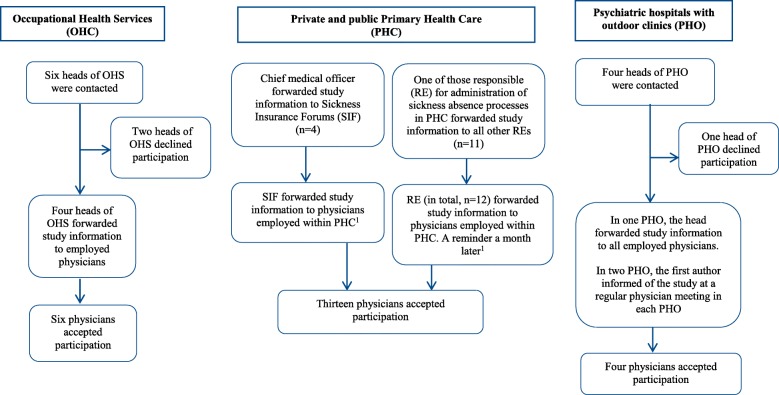
Description of the recruitment of participants from the Occupational Health Service (OHS), Primary Health Care (PHC) and Psychiatric hospitals with outdoor clinics (PHO). The geographical recruitment area was the Region Västra Götaland, Sweden. Twenty-three participants were recruited from this scheme; one participant was recruited after the final pre-interview (*n* = 24). ^1.^No information whether any SIF or RE declined to forward the study information

Physicians specialized in general practice, occupational health or psychiatry, with experience of treating patients with depression and anxiety disorders were approached through different channels and received an invitation letter with information about the study (Fig. 1). All participants interviewed were also asked to forward invitation letters, which most participants agreed to do. These sampling methods comprise an inability to know the exact number of physicians who received study information and declined participation. Interested physicians (*n* = 24) contacted the first author. Twenty-one interviews took place at the participants’ work place and three interviews in university facilities. The characteristics of the participants are presented in Table 1. Twenty-one participants had received a brief education on sickness insurance of 1–5 days; three had received no specific sickness insurance education. Three participants had previously been medical consultants to the SSIA.

**Table 1:** Characteristics of participants

Characteristic	Number
Health care setting^a^	
Occupational health care	6
Primary health care	13
Psychiatric outpatient clinic	5
Geographic setting	
Big city	8
Smaller town/rural area	16
Gender	
Male	12
Female	12
Age (years)	
Range	42–69
Mean	55
Specialization^b^	
Primary health	15
Psychiatry	6
Occupational health	3
Rehabilitation medicine	2
Other	3
Years since medical degree	
Range	9–40
Mean	26
Years since specialization^c^	
Range	1.5–37
Mean	18

^a^At the time of the interview. Many participants had experiences from several health care settings

^b^Five participants had two specializations

^c^Since first specialization, *n* = 21; three participants did not state year of specialization, however they had 17, 28 and 36 years of experience since their medical degree

### Data collection

The interview was structured using an interview guide with open-ended questions and a video vignette. The interview guide was elaborated by MB, JL, GA and GH, based in the authors’ experiences from mental health, sickness absence and work capacity research. All, but JL, have clinical experiences, GA as a physician. The questions were designed to address tacit knowledge. The participants were encouraged to consider and verbalize how they assessed capacity to work for patients with depression and anxiety. The video vignette has been used in previous Nordic studies investigating how GP’s diagnose patients with severe subjective health complaints [31] and recommendations of sickness absence [32]. The video vignette was based on an authentic GP-patient consultation, but for anonymity reasons the patient was replaced by an actor who had seen the original film and acted in the same way as the patient did in the consultation. The patient was a woman, 35 years old, a teacher in primary school, with no previous sick-leave history or somatic/psychiatric disorder; she felt tired, does not get things done, was struggling, had sleeping disturbance, related her symptoms to work overload, had no other complaints; she felt she might need time off work. The vignette was 8 min long. The actors spoke Norwegian with Swedish subtitles [32].

Half of the interviews began with showing the video vignette followed by questions on what and how the participant would have assessed capacity to work and sickness absence in this case. The participants were then asked to describe their own procedure and actions when assessing capacity to work and sickness absence for real patients with depression and anxiety. They were encouraged to describe as much as possible how they assessed capacity to work, the kind of questions they asked, and their inner decision-making process. They were also asked to identify what they consider particularly important to include in the work capacity assessment of patients with depression and anxiety. They were encouraged to describe real cases/assessments, but confidentiality was maintained because they were told to not reveal any identifying patient information (Post vignette, see Additional file 1). The other interviews began with the open-ended questions on the participants’ own procedure in real cases. The video vignette was shown somewhere in the middle of the interview, followed by questions on what the participant would have done in such a case (Mid vignette, see Additional file 1). We presented the vignette at different times in the interviews to minimize possible effects of the patient’s gender on the participants’ statements. All interviews were performed by the first author (an occupational therapist, female and PhD, experienced in qualitative research and with long clinical experience in psychiatry). All interviews were audio-recorded except during the video vignette. Notes were taken during the interview. These were used by the interviewer to create probes to encourage thorough and deep descriptions of how the assessment of capacity to work was performed. The interviews took place during 2014 and 2015.

In the preparation for the interview setting, pre-interviews took place with two GPs. In particular, the video vignette was tested for appropriateness as stimuli. Both GPs found the vignette valuable. From the GPs comments, the interview guide was nuanced to more directly address physicians, and the presentation of the vignette was refined to connect better to study aim. The final interview guide and procedure were tested with one psychiatrist who previously worked in primary health care. Since no changes were made, the interview was included in the study. Of the 24 interview recordings, three interviews lasted <29 min, 12 interviews lasted 30–39 min and nine interviews lasted >40 min. All recordings were transcribed professionally. The transcriptions were compared with the audio-recordings by the first author, and any mistakes were corrected.

### Analysis

Content analysis with an inductive approach was used in the analysis. Systematic text condensation described by Malterud (2012) [33] guided the steps in the analysis. Malterud [33] emphasizes the benefit of creating “a wider analytic space”, therefore all but one researcher were included in the identification of meaning units. The researchers are experienced in various disciplines (e.g. medicine, psychology, occupational therapy, social medicine). Once all interviews were performed, the first author only listened to all the recordings. Then, all transcripts were read to get a sense of the whole, followed by identification of meaning units. The same procedure was used by the other researchers who analysed six transcripts each. All meaning units were then included in further analysis performed with NVIVO 10 software by the first author [34]. First, the meaning units were organized into preliminary constructs, which were further analyzed and refined into more nuanced constructs. In the next step, the constructs were analyzed and organized into preliminary categories. To strengthen credibility, the preliminary findings were presented at a seminar for physicians in Occupational Health Services who were familiar with the phenomenon investigated, three of whom were participating physicians in the study. Then, the authors met and thoroughly discussed the preliminary findings, the Occupational Health Service physicians’ reflections and further analyses. The final analysis identified categories and sub-categories. At that point, the translation into English began while also condensing the results.

## Results

The aim of this study was to explore what and how physicians do when they assess capacity to work and the need for sickness absence in patients with depression and anxiety disorders. Five categories in the process of assessment were identified (Table 2).

**Table 2:** The categories and sub-categories identified in the analysis

	Category				
	1	2	3	4	5
	Identifying, understanding, creating and fitting the pieces together in the work capacity jigsaw puzzle	The significance of the disorder while assessing work capacity and sickness absence	Identifying work-place-related pieces of information	Identifying capacity in everyday life; contextual pieces of information	Assessing the need for sickness absence
Sub-categories	a. Using previously acquired personal experiences		a. Identifying work setting, work tasks and work demands		a. Issuing sickness absence in cases of decreased work capacity
	b. Sharpening the prime source of information: the patient		b. Identifying potential risk situations at work		b. Using sickness absence as a tool
			c. Understanding the patient at work		

### Category 1: Identifying, understanding, creating and fitting the pieces together in a work capacity jigsaw puzzle

The physicians did not describe a formalized work capacity assessment procedure. Instead, they described it as doing a “jigsaw puzzle” without any master model, using both tacit and explicit knowledge. The physicians needed to both identify and create the pieces, mainly from information supplied by the patient. An essential part of the jigsaw puzzle was the physicians’ correct understanding of a large amount of diverse information, representing very different characteristics, which was managed and operated during the physician-patient encounter.

It’s like doing a jigsaw puzzle, sometimes is it easy and sometimes quite difficult. You make the assessment based on their difficulties, [trying to understand] what kind of limitations do they really have? (Interview 5)

The finalized jigsaw puzzle was considered to be a comprehensive picture and required for adequate assessment of work capacity and need for sickness absence; however, minor assessments were constantly performed while identifying, creating and fitting the pieces together. They stressed that the jigsaw puzzle was highly individualized, which was the reason why patients who appeared to be alike (e.g. same disorder) were assessed differently. An essential prerequisite was time because identifying, understanding, creating and fitting the pieces together were time consuming. Although the participants described work capacity assessment, they also closely linked it to clinical reasoning. Jigsaw puzzling relied on two distinctive features, identified as two subcategories: “Using previously acquired personal experiences” and “sharpening the prime source of information – the patient”.

#### Sub-category 1a: Using previously acquired personal experiences

The participants described how they used experiences acquired from previous cases and seemed to compare present cases to earlier ones as an aid in the work capacity jigsaw. A difficulty with work capacity assessment was understanding the patients’ work demands and work settings, and how these were associated with the patients’ work capacity. They described how they used private experiences of different work settings, e.g. the health care context or jobs before being a physician; they described the health care setting as the one setting they were really knowledgeable of and therefore competent to understand. All these experiences built up a kind of knowledge bank from which they used information to understand the patient’s situation. Some participants had done field studies in large work places in their catchment area to improve their understanding of certain work settings.

… then I could ask, as I did yesterday to a patient, she is a geologist and she explained to me that she install flowmeters in wells and bring numbers back to the office for modelling where they need to take care of the rainwater in order avoid flooding elsewhere. Since I have built a house myself, I can understand such things as drainage and flows and water levels and so on. So I let her describe a project for me, and then I realized that she was having a hard time concentrating and getting tired very easily, making it difficult to perform because she could not think clearly. (Interview 16)

#### Sub-category 1b: Sharpening the prime source of information: The patient

Of particular importance for the work capacity jigsaw puzzle was facilitating patients to better verbalize their work capacity; a detailed and rich patient story was considered a prerequisite for identifying and creating pieces of information. Therefore, the participants underscored the need for sufficient time for each patient encounter but also time for several appointments. The patients’ ability to account for and supply information was consequently essential. However, that ability varied among patients; some patients do not have the linguistic ability; others are not used to discussing and describing their work capacity. It was necessary to create trust in the physician-patient encounter and was considered to be a foundation for obtaining correct information. The participants also used the trustful encounter to explain about disorders, work capacity and sickness absence, and regarded that as facilitators for the assessment.

I try to get them [the patients] to verbalize, and that is the hardest part for the patient because they have perceived they cannot manage the work, but it is very difficult for them to describe in what way. (Interview 15)

The participants stressed the importance of letting patients speak in their own words, a strategy that facilitates the patients’ own understanding of their situation, creating better informers. Open-ended questions and follow-up questions were used to help the patients with their story telling. The participants constantly asked the patients to make the story and descriptions clearer. Another strategy was to investigate work capacity over time. The participants asked the patients to clarify how it used to be (as a reference point), how it was now, and what was perceived to have changed; and was there any fluctuation in work capacity during the work day. The participants also used complementary informers, such as patients’ relatives or supervisors, who were expected to contribute with a more objective picture. For the same purpose, patients could also be asked to explain what other people meant about the patient’s capacity. The participants stressed the importance of not creating incorrect and subjective understanding of the patient’s work capacity.

I ask the patient to tell me more, to be more specific, to tell from your [the patient’s] point of view so I don’t place pictures of my own pre-understanding about what they [patients] do. I do not ask [precise questions] rather I say; tell me more, what are your work tasks? Open questions so that I get a picture of how it looks. (Interview 2)

In addition, the participants wanted the patients to disclose their perceptions of seriousness, opinions about sickness absence duration and/or possible solutions in order to maintain work. Such information was considered an important marker of how realistic the patients were and was also used for the participants’ clinical reasoning.

I always ask the patient, how long should we state [the sickness absence duration]? Just that, by asking the patient, do you have any suggestion, he discloses for me his own views. That have helped me many times, because sometimes they are too narrow in their time conception and reckon they will return to work sooner. It could also be the opposite, if the patient says, I need to be sick-listed for three months, well then I know that the motivation to return to work is very low. (Interview 24)

### Category 2: The significance of the disorder when assessing work capacity and sickness absence

All participants included the diagnostic and medical treatment procedure in the work capacity jigsaw puzzle. Knowledge of the extent and severity of the CMDs was essential because that determined to what extent the capacity to work would be affected. A common scenario was that the patient’s mental health problems was hidden behind somatic symptoms. Thus, the participants emphasized the importance of a correctly diagnosed CMD and any comorbidity. Comorbidity was considered to be common in these patients, mainly musculoskeletal disorders. An important sign of both the disorder (depression in particular) and decreased work capacity was the patient’s description of no longer recognizing themselves or their own work performance. Many participants stated that they deliberately asked patients “do you recognize yourself and your work performance or do you perceive you are another kind of person than you used to be”, because that perception was considered to be a distinctive description for these disorders and for decreased work capacity. Knowledge of earlier CMDs was an important source of information regarding the prognosis for work capacity. The participants also emphasized their responsibility for the medical treatment, which needs to be accounted for and balanced in relation to the work capacity and sickness absence assessment. They had to consider both the full effect and any potential side effects such as reduced alertness, decreased reactions or increased anxiety. The participants claimed that such issues must be considered before any reasonable assessment of work capacity can be made.

There is a disorder we call generalized anxiety disorder, I mean GAD, which naturally could be very troubling for some and there you simply need to consider the depth of the sickness. In a serious case of GAD, well, then the patient might not work at all because that would be at the cost of being stoned [due to medication] and that on the other hand is unfit with doing work. (Interview 13)

A specific dilemma in the assessment was that patients seldom have a continuously decreased work capacity throughout the day. The work capacity varies in relation to the symptoms (e.g. anxiety, increasing tiredness). Anxiety in particular was highlighted, because the patient could have capacity to work in one place (feeling safe) but not in another (feeling unsafe). Another difficulty was that between anxiety attacks, the patient can work as normal, but while having an anxiety attack, work capacity was affected instantaneously. Particular medical issues thought to be important in work capacity assessment are shown in Table 3.

**Table 3:** Areas/questions of importance in relation to medical issues, as described by physicians, for work capacity assessments of patients with common mental disorders, (category 2)

Medical dimensions:	
–	if the patient recognizes him/herself as the kind of person they used to be
–	if the patient recognizes their work performance as it used to be
–	sleeping problems
–	alcohol and/or other addictions (affects both disorders and functions)
–	suicidality
–	any triggers at work or home maintaining disorders
–	how symptoms affect functioning
–	variation in circadian rhythm

### Category 3: Identifying work place-related pieces of information

Several dimensions at work were considered essential pieces of the jigsaw puzzle to understand and assess a patient’s work capacity. Three subcategories were identified, related to the work place, safety and the patient him/herself.

#### Subcategory 3a: Identifying the work setting, work tasks and work demands

The content and demands of the work tasks were of major importance to understand the nature of the patient’s work. The participants described specific question areas that facilitated the identification of vital information (Table 4). These were thought to contribute to an understanding of the patient’s present work and what the patient was doing during the day. By simultaneously using the strategies described in sub-category 1b, the participants got a comprehensive picture of the patient’s present work performance.

First I ask about the history; have you experienced any difficulties in your work earlier? It could be a person working for 10, 20 years [in health care] saying, ‘I have never had any troubles encountering patients but now I don’t dare do that any longer.’ Then I [the physician] can establish that this person has a fundamental capability to encounter patients in crisis. (Interview 12)

**Table 4:** Areas/questions of importance in relation to the work place, as described by physicians, for work capacity assessments of patients with common mental disorders, (category 3: sub-category 3a and 3c)

**Sub-category 3a: work setting, work tasks and work demands:**	
Type of occupation	
Form of employment (permanent, limited, other)	
Any recent work changes (reorganization, new duties/routines/responsibilities, new manager/colleague)	
Amount of influence and decision making in own work situation	
Timely issues:	
–	working time (hours/week, hours/day, shift work, overtime)
–	work day breaks, possibilities to take breaks, avoiding breaks (why)
–	adherence to times, work speed demands, keeping up the pace of the work
Type of work tasks and inherent demands:	
–	simple or complex tasks
–	physical demands
–	mental demands (concentration, memory, endure stress, planning, multitasking, understanding information, communication, expressing oneself)
–	emotional demands (being alert, interactions and cooperation with other people, handle other peoples’ emotional reactions such as customers, pupils, clients)
Work environment:	
–	amount of surrounding stimuli and sounds
–	working alone or in group settings
–	often interrupted or disturbed
–	amount of support from colleagues/manager
Earlier work capacity (present work, earlier work)	
Any work accommodations: what kind of adjustments	
**Sub-category 3c: the patient him/herself:**	
–	formal and informal roles (e.g. being the most experienced/longest employed and therefore often asked questions)
–	likes his/her job and work tasks
–	work motivation in both actual and earlier jobs
–	earlier work experiences (including reasons for leaving/changing jobs)
–	having education/qualification for the job, feeling comfortable with work tasks
–	relationship with colleagues, manager (supportive, reliance, unfriendly, conflicts)
–	bullying
–	sexual harassment or assault at work
–	what has the patient told the manager/colleagues about his/her work problems and responses to that
–	in the patient’s opinion, what does manager/colleagues think of the patient’s work performance (trust/complaints)

#### Sub-category 3b: Identifying potential risk situations at work

An important dimension was to assess whether the decreased work capacity could lead to failures or accidents at work, in particular the risk of harm to oneself or third parties such as clients or customers, but also the risk of destroying machinery or products. The participants referred to the symptoms of CMD (e.g. tiredness, anxiety attacks), decreased functions (e.g. concentration difficulties) and the side effects of medications (e.g. decreased reactions) as risk factors. Example were chauffeurs, health care personnel (e.g. dealing with medicine, keeping patient records) and industrial or construction workers at heights where potential risks brought about the need for sickness absence.

The ability to react is often decreased; so if the patient drives a car as a work task, you need to consider that, because that could imply that they not are fit to drive a lorry or taxi or another kind of commercial vehicle as long as they experience decreased ability to react. That could be fatal, not just for the person him/herself. (Interview 18)

#### Sub-category 3c: Understanding the patient at work

The participants found it essential to understand what kind of a person the patient is at work and the patient’s perception of his/her work (Table 4). Two questions were particularly emphasized. First, whether the patient had the qualifications needed for the work tasks. Based on the participants’ experience, it was important to identify lack of qualifications because it could be confused with work incapacity and erroneously addressed with sickness certification. Second, whether the patient liked his/her job or not. The participants connected job satisfaction with the patients’ ability to endure work and considered it a factor that probably facilitated return to work.

### Category 4: Identifying capacity in everyday life: Contextual pieces of information

When assessing work capacity, all participants also included pieces of information about the patients’ capacity to manage daily tasks in their everyday life outside work, including both support and stress in the private situation. Handling everyday life satisfactorily was considered a prerequisite for managing work life. Exhaustion after a day’s work and the amount of recovery needed, e.g. hours needed for rest or sleep, were of particular importance (Table 5).

**Table 5:** Areas/questions of importance in relation to the patient’s everyday life, as described by physicians, for work capacity assessments of patients with common mental disorders, (category 4)

Factors and support/stressors in private life:	
–	family situation (single, married, divorced, children)
–	relatives in need of support (sick, old-aged)
–	anyone else in the family sick-listed or unemployed
–	support or not from husband/wife, relatives, others
–	financial issues
–	childhood and adolescence
Capacity to plan and manage home duties:	
–	taking care of family and children
–	shopping, cleaning, cooking, paying bills
–	reading and understanding newspapers, keeping up with news
–	watching and understanding television programmes
–	driving a car
Capacity to plan and carry out activities outside the home:	
–	exercising
–	hobbies
–	socializing with friends

### Category 5: Assessing the need for sickness absence

In cases where work capacity was assessed as decreased, the participants usually certified sickness absence. However, work place issues could influence the assessment otherwise. Sickness absence was also considered a risk for not returning to work, therefore the certification could be linked to return to work planning from the very beginning. Only a few participants explicitly described how they discussed the risks of sickness absence (e.g. long-term sickness absence) with their patients, in the same way that they discuss other potential risks with interventions. The participants also certified sickness absence for reasons other than decreased work capacity. Two sub-categories were identified: “issuing sickness absence in cases of decreased work capacity” and “using sickness absence as a tool”.

#### Sub-category 5a: Issuing sickness absence certification in cases of decreased work capacity

The participants had very different views regarding the extent (full-time/part-time) and duration of sickness absence. From the pieces of information described earlier (in earlier categories), the participants stressed the risks for accidents at work and the need for several hours of rest or sleep after a work day as very strong reasons for certifying sickness absence. Moreover, the possibility of a patient making a fool of him/herself or doing a job poorly was described as a reason for sickness absence, because this was seen as a potential obstacle in rehabilitation or return to work, and sickness absence was considered a preventive action in these cases. Participants also more easily certified sickness absence in cases where work place adjustments had been made and the patient still had difficulties managing work, or in cases where managers could not make work adjustments.

She [the patient in the vignette] is quite ill and she might lose face by exposing her at work. She has a hard time holding herself together and she has to perform in a working team. She is quite exposed as a teacher in front of the students. The issue is also about protecting people while they suffer from the mental illness so they don’t get hurt and can manage to return to their work again; well she has that kind of a work. However, it could also be persons in managerial positions where employees absolutely should not see them  while they are sick. Because then they cannot return to a position with authority. (Interview 2)

In addition to decreased work capacity, the participants also took into account their perception of the patient’s work place in the assessment of the need for sickness absence. In well-managed work places, the participants’ reasoned that patients were able to work despite decreased work capacity. However, when they perceived the work place as dysfunctional, the participants were more likely to see the need for sickness absence. In cases of work place conflicts, the participants believed such things needed to be dealt with at the work place and sickness absence should be avoided.

#### Sub-category 5b: Using sickness absence as a tool

Sickness absence was often described as a means to having enough time for a thorough diagnostic procedure and assessment of work capacity, but also having time to await recovery and/or the effect of medication were stressed as prerequisites for a thorough work capacity assessment. A third scenario was issuing sickness absence to prevent deterioration of the disorder; although not strictly speaking part of work capacity assessment, the participants linked them closely.

At the first visit, I try to find out as much as possible about the work and the network and so on. The next time, I go deeper trying to figure out what has happened. I try to get further information and also to see the patient in another stage, how is she functioning at this time, if she is still as closed off, or whatever it might be [as the first visit]. (Interview 18)

## Discussion

To our knowledge, this is the first study aiming to get an understanding of physicians’ tacit knowledge of what and how they assess work capacity and need for sickness absence in patients with CMDs. The assessment was described as doing a jigsaw puzzle where the physicians themselves needed to identify, understand, create and fit pieces of very diverse information about the patients’ disorder, work setting and everyday life together in a comprehensive picture. The assessment was a really complex procedure. The findings show that these physicians used both a biomedical approach through medical procedures and a holistic approach where they used a broad palette of information about the person, the environment (including both the work and home environment) and tasks/occupations at work. The latter approach is in line with the Person-Environment-Occupation (PEO) model [35].

The PEO model states that occupational performance can be understood as the interplay between the person, the environment and the tasks the person is to pursue, and in particular, there is a dynamic ongoing process between the parts [35]. Support or barriers in any one of these parts could promote or hamper the capacity to work. The participants’ use of information about patients’ fluctuating work capacity, as well as symptoms during the day, and in different settings, show how their practice relates to the dynamic aspect in the PEO model. The holistic approach in our results is in line with the findings of Foley et al. (2003) [13] where physicians investigated workload, relationships at work and social networks when assessing fitness for work. Furthermore, they inquired more about social and private conditions in cases with psychological problems compared with physical problems, corroborating our findings [13]. However, investigation of work tasks was an important part of the assessment in our study, contrary to Foley et al. (2013) [13], where work tasks were less investigated in cases with psychological problems. The description of work capacity assessment as a jigsaw puzzle, complex in its nature, has also been shown in quotes from physicians elsewhere [7].

Unlike an ordinary jigsaw puzzle, our participants did not have a box of pieces to use. No participant used a formalized work capacity assessment to identify the pieces of the puzzle, which is in line with earlier findings [17, 36]. Instead, participants emphasized the physician-patient encounter. Creating trust and using a communicative style that allows knowledgeable information to emerge and be identified seemed to be a cornerstone in the jigsaw puzzling. Here, the physicians used tacit knowledge, obtained through several encounters and also personal experiences from professional and private settings. In addition, they used explicit knowledge (e.g. medical procedures, record information) and in particular they underscored the importance of enough time for the consultation. Time constraints as a barrier for doing adequate assessments have been confirmed in several studies [3]. As a consequence, Bremander et al. (2012) [37] found that physicians without the possibility to extend the consultation time when necessary issued sickness certificates more often. A further complication in these assessments was the patient’s changing “present”, because greater understanding of the patients work capacity over time, and the natural course of recovery or deterioration in patients, contributed to changed, added or vanished puzzle pieces over time.

The sub-category “sharpening the prime source of information – the patient” showing patients’ inadequacy as informers as a result of unawareness or unaccustomedness is an angle that has not been described previously. Earlier studies have reported that subjective patient stories provide the basis for work capacity assessments, which physicians have experienced as dissatisfying [7, 8, 17]. The strategies described to overcome the patients’ inadequacies in conjunction with questions from the other categories (category 3 in particular) can improve the quality of the patient stories and subsequently the assessments. Byrne et al. (2014) identified several strategies used by physicians while negotiating with patients, and among them, the two strategies “fact finding: occupation” and “information gathering” were in line with our findings [38]. The present study contributes with more details of the information gathering process. Physicians could also gain useful knowledge from the few studies on the phenomenology of work capacity in individuals with CMD [23, 24] to further improve their supporting strategies. The patient’s gender did not appear to be an issue in the work capacity assessment. That could have been expected considering that women are affected by CMD two to three times more frequently than men [39], and the higher sickness absence rates reported among women [40]. Another qualitative study reported that health care professionals perceived work capacity to be affected in similar ways in men and women [23].

One important finding, and to our knowledge not described previously in relation to CMD, was the physicians’ safety concerns for the patient but also for third parties or the work place. This subject is seldom mentioned in sickness absence and return to work studies. In the field of functional capacity evaluations (FCE), it has been argued that “FCE is performance based measurement to determine what the person *can do safely*, not what he/she *can’t do*” [41] (italics by Soer et al.). Although these safety issues were mainly related to the FCE procedure, they still highlight the importance of being able to do one’s work safely. In Gärtner et al.’s (2010) [42] review on the impact of CMD on work functioning in health care workers, they found that health care workers with CMD made more errors than their healthy colleagues, which among other things endangered patient safety. These findings corroborate with the physicians’ safety concerns in our study, and the distinction between can work and can work safely seems to be a crucial issue. In our findings, the safety concerns mainly related to physical consequences. However, in work duties involving concluding agreements or making business deals, there could be other far-reaching consequences for both the person and the employer. One consideration in relation to patients with CMD is the risk of stigmatizing attitudes towards these people in the work place [43]. Addressing safety matters might lead to increased stigma; however, according to the findings of Gärtner et al. [42] and our findings, it still seems ethically important to do this. We also found physicians in our study considered “soft” safety concerns aimed at making sure that the patient did not make a fool of him or herself at work.

In the category of “assessing the need for sickness absence”, the statements on the extent or duration of sickness absence were found to be in line with other studies [32]. Most participants thought of sickness absence as a risky intervention, corroborating Macdonald et al.’s (2012) [4] qualitative study and their category “sickness certificates: a powerful intervention” in which physicians considered sickness certification as a risky tool because of its potential counter effects. However, based on our participants’ tacit knowledge of how and what they do, only a few participants explicitly described discussing the risks of certifying sickness absence with their patients. That non-action might be interpreted from a clinical reasoning point of view whereby the patient’s recovery or risk of further deterioration is at the forefront of the physicians’ decision making. Sickness certification is then used as a protective tool (shown in sub-category 5b), which hinders a discussion of risks. To the number of studies reporting sickness absence negotiations between the physician and the patient [14, 38] this study adds that physicians negotiate, or reason, with themselves using their apprehensions of the patients’ work environment as an important piece of understanding while assessing work capacity. Thus, they dismiss the individual patient’s capacity to work as the sole ground for decisions.

### Implications for practice

The complexities and difficulties of assessment of work capacity have been identified in several studies [2, 3, 5, 6]. However, earlier studies report inadequate training for physicians in these matters in many countries [3, 16]. Contemporary education has also been perceived inefficient in relation to work capacity assessment [16], and has not been found to decrease sickness absence [44]. In addition, one large Swedish study found that informal learning situations between colleagues were the most reported path of knowledge among physicians [16]. Such knowledge distribution is indeed transference of tacit knowledge. In our study, three participants reported no education, and the others only brief education of a few days. All had long clinical experience, however.

Improvement in learning of work capacity assessments and insurance medicine in general has been neglected for a long time. This needs to be prioritized given the large impact on society, work life and patients on long periods of sickness absence in particular. It is necessary to balance the benefits of adequate sickness insurance schemes (e.g. recovery from disease) against the risk of large economic drawbacks (e.g. societal costs and the risk of long-term or permanent exclusion from the labour market). The physicians need transformation of tacit knowledge into explicit knowledge, complemented by existing knowledge in this field in other disciplines. Improving formal education from these different sources might better fit into the physicians’ curriculum and be implemented more easily into contemporary work. At least it would increase physicians’ awareness of the importance of their role in the sick listing process.

The five categories in the assessment of sick-listing patients identified in this study could be used systematically by physicians in their daily practice to ensure that in every case the assessment takes all these steps into account. Future studies could explore whether such a strategy would ease or at least standardize the assessments, and bring the tacit knowledge into explicit knowledge. Several studies have shown that physicians experience difficulties in negotiating with patients concerning sick-leave [14, 15, 38], our study can contribute to better equip less experienced physicians to improve their assessments and facilitate the sick-leave decisions.

### Methodological considerations

We strived for tacit knowledge, because we believed the assessment task to be embedded in physicians’ clinical practice and not fully consciously made. However, the analysis revealed that both tacit and explicit knowledge was at hand. Should we then have left the explicit knowledge out of the study? We consider the tacit and the explicit knowledge to be so intertwined that leaving out the explicit knowledge (e.g. medical procedures) would violate the validity of the study. In particular, the study would not have been truthful to the participants’ clinical practice of assessing work capacity. Another important methodological issue is the selection of the participants. It is probable that we mainly reached physicians with a specific interest in work capacity and sickness absence. For the aim of this study, we do not consider that as a limitation, because it is likely that their interest supported their conscious thought about what and how they were doing. More problematic is whether the data are invalidated by desirability, have the participants really told us what they did, or rather what they think they did or what they want to do. From the many patient cases discussed, we do believe we have valid data; however, the participants did not execute an exhaustive assessment with each patient. To investigate actual clinical practice, a study of recordings and analyses of physician-patient sickness absence encounters would provide a great deal of information.

The video vignette was used as a stimulus and several participants claimed it to be a realistic case. We played it at different time during the interview (first and in the middle) to avoid the particularities of the vignette affecting the results of the study. Although the vignette has been used in several studies, also in Swedish settings, some participants commented on the physician’s attitude. These participants considered the physician in the vignette was not client centred and used inappropriate leading questions. Our participants stressed the importance of open-ended questions, however the strong emphasis on that strategy might be due to a reaction to the physician in the vignette. Moreover, health care policy in Sweden have advocated a client centered and participatory care in which open-ended questions are an important communicative tool.

The participants were of both sexes, different ages, and from different geographic areas, including large and small towns and rural areas; they met a broad variety of patients, which contributed to the trustworthiness of the findings but also to more general transferability of the results [45]. Involving several authors in identifying the meaning units increased the credibility of the findings [30, 45]. All authors were knowledgeable of qualitative methodology and were from different disciplinary and professional backgrounds. That combined pre-understanding contributed to reflexivity during the analysis and the trustworthiness of the study [30, 45].

## Conclusions

In this study, physicians’ tacit knowledge of assessing work capacity and the need for sickness absence for patients with CMDs was identified as doing a jigsaw puzzle. The physicians became creators of the pieces of the puzzle and used a broad palette of information about the person, the environment (including both the work and home environment) and tasks/occupations at work. Because patients were considered the prime source of information, it was important to facilitate strategies to enhance the patients’ capacity to better communicate the essential information as well as for the physicians own decision making. It was of major importance to assess the patients’ capacity to work safely and not endanger themselves or a third party. Our findings based on physicians’ tacit knowledge provide physicians with the particulars needed for work capacity assessment. Applying these findings to formal education and training would contribute to improvement in physicians’ assessment skills, result in a better fit into the physicians’ curriculum and be implemented more easily into contemporary work.

## Additional file

Additional file 1: Interview guides: Post and Mid vignette. Interview guides used in interviews with physicians in the study “The capacity to work puzzle”. (DOCX 23 kb)

## Abbrevations

CMD: Common Mental Disorder
GP: General Practitioners
FCE: Functional Capacity Evaluations
OHC: Occupational Health Services
PEO: Person Environment Occupation Model
PHC: Private and Public Primary Health Care
PHO: Psychiatric hospitals with outdoor clinics
RE: Responsible personnel for administration of sickness absence processes in Primary Health Care, Region of Västra Götaland
SIF: Sickness Insurance Forums in Primary Health Care, the Region Västra Götaland
SSIA: the Swedish Social Insurance Agency
