# Supplementary material for: The capacity to work puzzle: a qualitative study of physicians’ assessments for patients with common mental disorders
Source: BMC Fam Pract. 2018 Jul 30;19:133. doi: 10.1186/s12875-018-0815-5 (PMC6066915; doi:10.1186/s12875-018-0815-5)
Supplement: Supplementary file 1 — Interview guides: Post and Mid vignette. Interview guides used in interviews with physicians in the study “The capacity to work puzzle”. (DOCX 23 kb) [file 12875_2018_815_MOESM1_ESM.docx]

**Additional file**

**Interview guide Post and Mid vignette**

**A: Interview guide Post vignette**

1. The Vignette
2. Departing from this case and scenario, how would you proceed to assess capacity to work and sickness absence?
3. Departing from your own clinical cases, how do you usually proceed when you assess capacity to work, can you describe your proceedings and your inner decision-making process.
4. What do you think is important to ask for and to find out to be able to assess capacity to work?
5. What do you think is important to include in the assessment of capacity to work in patients with depression and anxiety?
6. Is there anything specific that you find particularly important to find out in relation to patients with depression and anxiety?
7. How do you usually ask to find out the information you consider important in order to assess capacity to work?

Probes:

- How do you do then?
- What do you ask?
- How do you get that?
- What makes you ask about that?

**B. Interview guide Mid Vignette:**

1. Departing from your own clinical cases, how do you usually proceed when you assess capacity to work, can you describe your proceedings and your inner decision-making process.
2. What makes you ask about these particular things?
3. How do you usually ask for things you need to know?
4. What do you think is important to ask for and to find out to be able to assess capacity to work?
5. Is there anything specific that you find particularly important to find out in relation to patients with depression and anxiety?
6. The vignette.
7. Departing from this case and scenario, how would you proceed to assess capacity to work and sickness absence?
8. What would you have done if you had met this patient?

Probes:

- How do you do then?
- What do you ask?
- How do you get that?
- What makes you ask about that?
